# The Effects of Overfeeding on the Neuronal Response to Visual Food Cues in Thin and Reduced-Obese Individuals

**DOI:** 10.1371/journal.pone.0006310

**Published:** 2009-07-28

**Authors:** Marc-Andre Cornier, Andrea K. Salzberg, Dawnielle C. Endly, Daniel H. Bessesen, Donald C. Rojas, Jason R. Tregellas

**Affiliations:** 1 Division of Endocrinology, Metabolism, and Diabetes, Department of Medicine, University of Colorado Denver, Aurora, Colorado, United States of America; 2 Department of Psychiatry, University of Colorado Denver, Aurora, Colorado, United States of America; 3 Department of Medicine, Denver Health Medical Center, Denver, Colorado, United States of America; 4 Research Service, Denver VA Medical Center, Denver, Colorado, United States of America; University of Parma, Italy

## Abstract

**Background:**

The regulation of energy intake is a complex process involving the integration of homeostatic signals and both internal and external sensory inputs. The objective of this study was to examine the effects of short-term overfeeding on the neuronal response to food-related visual stimuli in individuals prone and resistant to weight gain.

**Methodology/Principal Findings:**

22 thin and 19 reduced-obese (RO) individuals were studied. Functional magnetic resonance imaging (fMRI) was performed in the fasted state after two days of eucaloric energy intake and after two days of 30% overfeeding in a counterbalanced design. fMRI was performed while subjects viewed images of foods of high hedonic value and neutral non-food objects. In the eucaloric state, food as compared to non-food images elicited significantly greater activation of insula and inferior visual cortex in thin as compared to RO individuals. Two days of overfeeding led to significant attenuation of not only insula and visual cortex responses but also of hypothalamus response in thin as compared to RO individuals.

**Conclusions/Significance:**

These findings emphasize the important role of food-related visual cues in ingestive behavior and suggest that there are important phenotypic differences in the interactions between external visual sensory inputs, energy balance status, and brain regions involved in the regulation of energy intake. Furthermore, alterations in the neuronal response to food cues may relate to the propensity to gain weight.

## Introduction

The prevalence of obesity has risen dramatically in the United States over the last 30 years. While genes undoubtedly play an important role in the development of obesity, genetic influences would not be expected to change over such a short period of time. This suggests that environmental influences are likely playing a significant role in the cause of this epidemic and that interactions between relevant genes and environmental factors probably produce the obese state [1], [2]. One of the most dramatic changes in the environment over the last 40 years has been the broad availability of relatively inexpensive highly palatable food [3]. It is likely that most individuals experience periods of positive energy balance when exposed to the modern western diet [4]. Why then do not all people when exposed to highly palatable food eat in excess and become progressively more obese? An individual's susceptibility to weight gain and obesity may relate to the capacity of that individual to sense and respond appropriately to these periods of positive energy balance. Those, for example, who are genetically predisposed to thinness in the current environment may be able to sense and respond to excess energy intake more rapidly and accurately than those predisposed to obesity [5], [6].

The regulation of energy intake is a complex process requiring the integration of multiple internal as well as external signals. A great deal has been learned about the homeostatic regulation of energy balance and the effects of adiposity and gut signals on hunger and satiety [7], [8]. Ultimately, however, the decision to initiate food intake, how much to consume, and when to terminate a meal is affected by not only these homeostatic mechanisms but also by learned behaviors, cognitive factors, habits, social context, availability of food, and external sensory cues and the integration of these different sensory inputs [9]. It has been hypothesized that the regulation of food intake follows the structure of motivated behavior [10]. First, visceral and external sensory inputs are processed and integrated with reward and memory systems leading to an “incentive value” of the goal. Behavior is then initiated following the interaction of the internal state, such as state of energy balance, and the incentive value of the goal, i.e. food. This motivated ingestive behavior is the outcome of the integration of stimulatory, inhibitory, and disinhibitory neural circuits. Once the behavior has been initiated, functions of reward and aversion, as well as learning and memory are critical in this integrative process.

We have begun to better understand the neural circuitry associated with the processes involved in ingestive behavior. A number of studies have examined the neuronal response to visual food cues in normal weight individuals. These studies have found a network of brain regions that are activated in response to visual food cues, including activation of prefrontal cortex, orbitofrontal cortex, inferior temporal cortex, insula, striatum, amygdala, hippocampus, and hypothalamus [6], [11], [12], [13], [14], [15], [16], [17], [18]. The salience of the stimulus also appears to be important with images of foods described as “of high hedonic value,” “high-calorie,” “appetizing,” or “fattening” resulting in differential activation of specific brain regions [6], [12], [16], [18], [19]. It is not known, however, whether the obese or reduced-obese states are associated with differences in neuronal responses to food cues as compared to normal weight individuals. It is also unclear whether short periods of excess energy intake impact the neuronal response to food cues differently in obese or reduced-obese individuals.

We thus hypothesized that thin individuals, individuals who “adapt” effectively to periods of positive energy balance, would be sensitive to food-related visual stimuli and that responses to these stimuli would be attenuated following overfeeding when the internal milieu should promote reduced food intake. In contrast, we hypothesized that reduced-obese individuals, individuals at high risk for weight gain, would be less sensitive to positive energy balance as seen by persistent neuronal responses to food-related visual cues despite overfeeding. We elected to only study reduced-obese and not obese individuals because we were most interested in studying individuals at risk for weight gain and not those who were already obese. The present study was designed to examine these hypotheses.

## Methods

### Ethics Statement

This study was conducted according to the principles expressed in the Declaration of Helsinki. The study was approved by the Colorado Multiple Institutional Review Board (02-585). All patients provided written informed consent for the collection of samples and subsequent analysis.

### Subjects

Thin (BMI 19–23 kg/m^2^) and overweight/obese (BMI 27–32 kg/m^2^) healthy, right-handed individuals aged 25–45 were recruited and screened. Thin subjects had no family history of obesity and were weight stable by self-report for greater than ten years. Eligible subjects were free of metabolic and psychiatric disease and eating disorders. Obese participants entered a weight loss program with a goal weight loss of 8–10% of their initial body weight. This was accomplished by on-going supervision and intervention by the University of Colorado Denver Clinical Translational Research Center (CTRC) research dieticians. The primary goal was weight loss and not a specific macronutrient composition or caloric prescription. Counseling was individualized in order to find the best plan for each participant. Once the weight-loss was achieved the reduced-obese (RO) subjects were maintained at this new reduced weight for 8 weeks prior to studies being performed. Individuals unable to achieve a minimum 5% weight loss and/or maintain their weight loss were excluded. Actual weight loss was 8.0±0.9% (mean±SD) of initial body weight. Twenty-two thin individuals (10 women, 12 men) and 19 weight-reduced individuals (10 women, 9 men) were studied (Table 1).

**Table 1:** Subject characteristics (number or mean±SD).

	Thin	Reduced-Obese
N (M/W)	22 (12/10)	19 (9/10)
Age (years)	34.4±5.1	35.5±5.7
BMI (kg/m^2^)	21.6±1.8	27.4±2.7*
Body Fat (%)	19.7±6.9	30.9±7.7*
Restraint	4.3±3.7	8.1±4.2*
Disinhibition	4.5±3.1	6.8±3.5*
Hunger	5.1±3.8	4.9±2.0

* p<0.05 for thin compared to reduced-obese.

### Study Design and Measurements

Subjects first underwent baseline assessments, including a 3-day diet diary, completion of the Three Factor Eating Inventory [20], measurements of resting metabolic rate (RMR) by hood indirect calorimetry (2900 metabolic cart, Sensormedics, Yorba Linda, CA), and body composition measurement by dual-energy x-ray absorptiometry (DPX whole-body scanner, Lunar Radiation Corp., Madison, WI). For the reduced-obese cohort these baseline assessments were performed in the reduced-obese state after weight maintenance.

Subjects were then studied on two occasions (eucaloric and overfeeding) in a randomized cross-over manner. In women, study periods were performed in the follicular phase of their menstrual cycle. At least 1 month separated the two feeding conditions. Each study period included a 3-day run-in diet phase and a 2-day “controlled” diet phase. The run-in diet phase was done to ensure energy and macronutrient balance. Estimates of daily energy needs were made using several factors: 1) usual intake via 3-day food diary, 2) the Harris-Benedict equation, 3) baseline RMR plus an activity factor, and 4) lean body mass. On one occasion (eucaloric), subjects were maintained on the eucaloric diet for 2 more days. On another occasion, subjects were overfed by 30% above eucaloric needs (overfeeding) for 2 days. The macronutrient compositions of the diets were the same in both conditions at 50% carbohydrate, 30% fat, and 20% protein. The saturated and poly-unsaturated fat ratio and fiber and cholesterol content of the diets were identical. All food was prepared and provided by the CTRC kitchen. Subjects presented to the CTRC every morning. They were weighed, ate breakfast, and picked up the remainder of their daily meals in coolers. They were asked to return any uneaten food, which was then measured and incorporated into their next day of food. Subjects were asked to maintain their usual pattern of physical activity and were regularly questioned regarding activity and compliance. Subjects were asked to not consume any alcoholic or calorie-containing beverages during the study period.

### Functional Magnetic Resonance Imaging (fMRI)

Subjects presented to the Brain Imaging Center at the University of Colorado Denver the morning after the second day of each controlled diet phase in the overnight fasted state. Imaging studies were performed using a GE 3.0 T MR scanner equipped with high speed gradients (300 µs rise time and maximum gradient strength 24 mT/m). Anatomical imaging was first performed. fMRI data were then acquired using EPI T2* BOLD (Blood Oxygen Level Dependent) contrast technique (TR = 2000,TE = 30, 64^2^ matrix, 240 mm^2^ FOV, 28 axial slices angled parallel to the planum sphenoidale, 4 mm thick, 0 mm gap). Functional imaging was performed while the subjects were presented visual stimuli using a projector and screen system. Visual stimuli consisted of three different categories: neutral nonfood objects (O), foods of high hedonic value (H), and foods of neutral hedonic or utilitarian value (U). Examples of O included images of animals, trees, books, furniture, and buildings. Examples of H included images of waffles with whipped cream and syrup, chocolate cake, cookies, plate of eggs and bacon, and pastries. Two runs each lasting 8 minutes were performed with each run consisting of a pseudo randomized block design with 8 blocks of pictures of H, 8 blocks of U, and 8 blocks of O. Each block consisted of 10 stimuli shown for 2 seconds each for a total of 20 seconds per block or 240 scans per run. Subjects were asked lie quietly and to view the images. The current analysis only examines the differences between the H and O stimuli. We have previously published data examining the differences between H and U stimuli in thin individuals [6]. Although the overall effects were qualitatively similar, the comparison of H to U in the present study was insufficiently powered to detect significant group differences. The H to O comparison was more robust than that between H to U, allowing for sufficient power to analyze the comparisons described here.

### Behavioral Measurements

Measures of appetite were done during each controlled diet period. Before and after each meal, subjects rated their hunger, fullness, and prospective consumption on visual analogue scales (VAS) as described by Rolls [21]. Hunger was rated on a 100-mm line preceded by the question, “How hungry do you feel right now?” and anchored by “not at all hungry” and “extremely hungry” on the right. Fullness was rated by the question, “How full do you feel right now?” with the anchors “not at all” and “extremely.” Prospective consumption was rated using the question, “How much food do you think you could eat right now?” anchored by “nothing at all” and “a large amount.”

### Calculations and Statistical Analyses

Functional images were analyzed with SPM5 (Wellcome Dept. of Imaging Neuroscience, London.). After discarding the first four scans from each run for saturation effects, images were motion corrected, normalized to standard space, spatially smoothed with a 6 mm FWHM kernel. After accounting for reslicing during preprocessing steps, the final smoothness of the data was approximately 3 times the acquisition voxel size. Data were then evaluated using the GLM in a random effects analysis. To generate the random effects model in SPM5, statistical parametric maps were first generated for each subject using the General Linear Model to describe the variability of the data on a voxel by voxel basis. Hypotheses expressed in terms of model parameters were assessed at each voxel with univariate statistics, yielding an image whose voxel values comprise a statistical parametric map [22]. The model consisted of an HRF-convolved boxcar function. Additionally, a 128 s high pass filter was applied to remove low-frequency fluctuation in the BOLD signal. A second level analysis was performed to incorporate both within subject and between subject variance, allowing inference to the population. Accordingly, each individual subject's data for each condition of interest, both within and across feeding conditions, were summarized with one parametric map (accounting for within subject variance), and then assessed across subjects (accounting for between subject variance), thereby implementing a random effects model . For the thin group alone, data were evaluated with a t-test. All other group comparisons and group by feeding condition interactions were evaluated with directional contrasts (SPM t-contrasts) in the context of a 2×2 repeated measures ANOVA. Data were corrected for multiple comparisons with the False Discovery Rate (FDR) technique, thresholding at q = 0.05.

In addition to the whole-brain analyses used to evaluate the main effects of stimulus type, region of interest (ROI) analyses were used to evaluate responses in less-powered comparisons, namely the effect of eating conditions, in the insula, hypothalamus, and inferior visual cortex. ROIs were defined anatomically and used for small volume corrections (SVC). Two ROI, the anterior short insular gyrus and fusiform gyrus, were derived from probabilistic labeling of the SPM single subject average image using Freesurfer (http://surfer.nmr.mgh.harvard.edu/). The probabilistic labeling was based on the Destrieux atlas of the Freesurfer package [23], and these two ROI included only the gray matter portion of the gyri. The ROI for the hypothalamus was generated from the Wake Forest University PickAtlas [24]. All ROIs were smoothed with a 4 mm FWHM kernel and intensity filtered at 0.5 to minimize artifactual increases in statistical thresholds due to large surface areas relative to volumes [25].

## Results

### Study Participants

Subject characteristics are summarized in Table 1. Despite losing approximately 8% of their body weight, the reduced-obese (RO) individuals had greater body mass index and percent body fat compared to the thin individuals. RO individuals had greater restraint and disinhibition scores on the Three-Factor Eating Questionnaire than thin individuals.

### Thin Individuals

**Figure 1:**
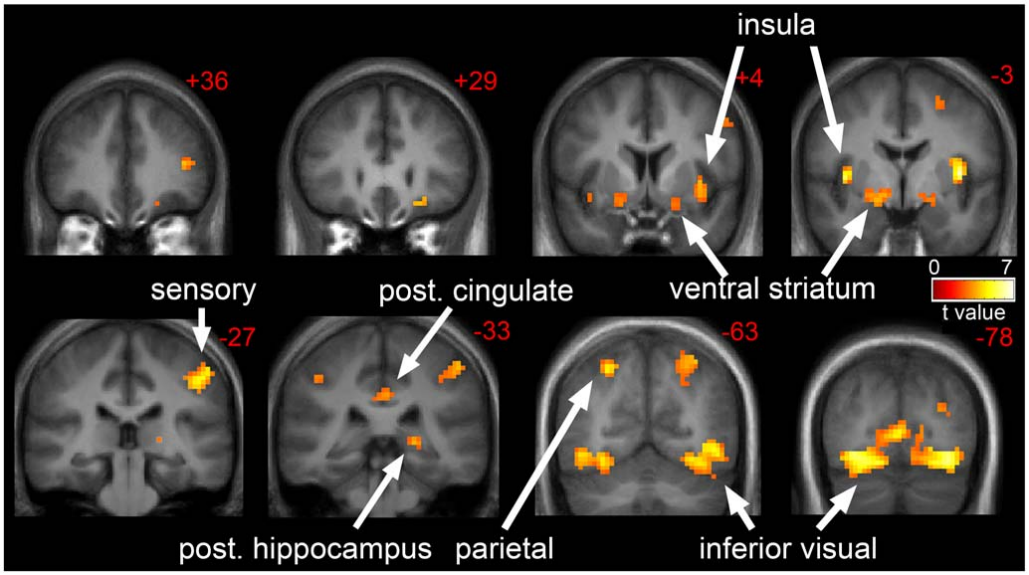
Neuronal response to visual foods cues in thin individuals in the eucaloric state. The neuronal response in thin individuals to visual stimuli of foods of high hedonic value as compared to non-food objects in the eucaloric state is shown (EU:H>O). Robust activation is observed in the insula, sensory cortex, posterior cingulate, ventral striatum, posterior hippocampus, parietal cortex, and inferior temporal visual cortex. Statistical maps thresholded at an FDR corrected threshold of q<0.05 and overlaid onto the group average anatomical image. Data are shown in the radiological convention (right hemisphere on the left).

First we describe the neuronal responses to visual stimuli as measured by fMRI in the thin cohort. In the “energy balance” or eucaloric state (EU), images of foods of high hedonic value (H) as compared to neutral nonfood objects (O) resulted in greater activation of insula, inferior temporal visual cortex, posterior parietal cortex, ventral striatum, inferior and middle frontal gyri, orbitofrontal cortex, posterior cingulate, posterior hippocampus, and sensory cortex (postcentral gyrus) as seen in Figure 1 and Table 2 (EU:H>O). A trend towards significant response in the hypothalamus was observed (t(1,21) = 2.86, p = 0.059). Two days of 30% overfeeding (OF) resulted in significant attenuation of the activation seen in the eucaloric state. Specifically, there was significantly reduced activation of the insula (t(1,21) = 2.74, p = 0.039) and hypothalamus (t(1,21) = 3.07, p = 0.029) with overfeeding as compared to eucaloric feeding (EU>OF:H>O). Overfeeding, however, did not result in greater activation of any brain regions as compared to the eucaloric state (OF>EU:H>O).

**Table 2:** Regions of increased neuronal activation in thin individuals in response to hedonic (H) compared to non-food (O) images, from whole-brain analyses in the eucaloric (EU) state.

*EU:H>O*	*Local maxima coordinates* *			*t value*	*p value*
	*x*	*y*	*z*		
Insula (L)	−39	−3	6	7.10	0.002
Insula (L)	−39	12	−9	4.05	0.010
Insula (L)	−36	15	−18	3.68	0.018
Insula (R)	39	−3	3	6.02	0.002
Insula (R)	39	6	−12	3.31	0.032
Inferior visual cortex (L)	−21	−84	−15	7.87	0.002
Inferior visual cortex (R)	36	−72	−15	6.71	0.002
Inferior visual cortex (R)	9	−90	−9	6.69	0.002
Parietal cortex (L)	−36	−51	60	5.78	0.002
Parietal cortex (R)	27	−72	33	5.46	0.002
Parietal cortex (R)	27	−60	48	5.35	0.002
Parietal cortex (R)	30	−54	54	4.28	0.007
Parietal cortex (R)	45	−36	42	3.94	0.012
Postcentral gyrus (L)	−48	−27	45	5.39	0.002
Postcentral gyrus (L)	−54	−30	51	5.13	0.002
Orbitofrontal cortex (L)	−21	33	−15	4.47	0.006
Orbitofrontal cortex (L)	−30	27	−21	3.08	0.038
Inferior frontal gyrus (L)	−39	36	12	4.26	0.008
Middle frontal gyrus (L)	−24	−9	54	3.67	0.018
Ventral Striatum (L)	−18	0	−12	3.49	0.024
Ventral Striatum (R)	18	−3	12	4.17	0.008
Hippocampus (L)	−21	−33	0	4.05	0.010
Cingulate gyrus (L)	−3	−33	33	3.71	0.017

* Stereotactic Coordinates in MNI space.

### Reduced-Obese Compared to Thin

**Figure 2:**
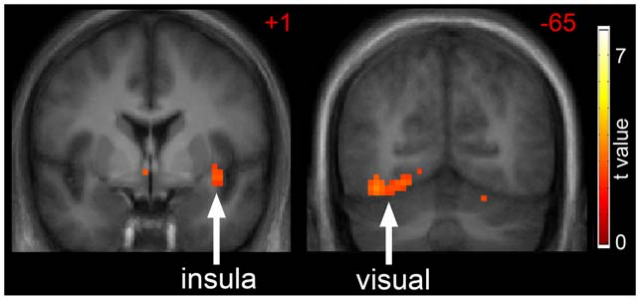
Neuronal response in thin as compared to reduced-obese individuals. The difference in neuronal response in thin as compared to reduced-obese individuals to foods of high hedonic value in the eucaloric state is shown (EU:Thin>RO:H>O). Greater activation of the insula and visual cortex is noted in thin as compared to reduced-obese individuals. Statistical maps thresholded at p<0.01 for visualization and overlaid onto the group average anatomical image. Data are shown in the radiological convention (right hemisphere on the left).

Next, we turn our attention to the RO cohort and how their responses differ from the thin individuals. In the eucaloric state, as shown in Figure 2, thin individuals had greater activation of inferior visual cortex (t(1,75) = 3.36, p = 0.044, right;) and a trend for greater activation of the insula (t(1,75) = 2.78, p = 0.097, left) compared to RO individuals (EU:Thin>RO:H>O). No brain regions were more activated in RO as compared to thin individuals in the eucaloric state (EU:RO>Thin:H>O).

**Figure 3:**
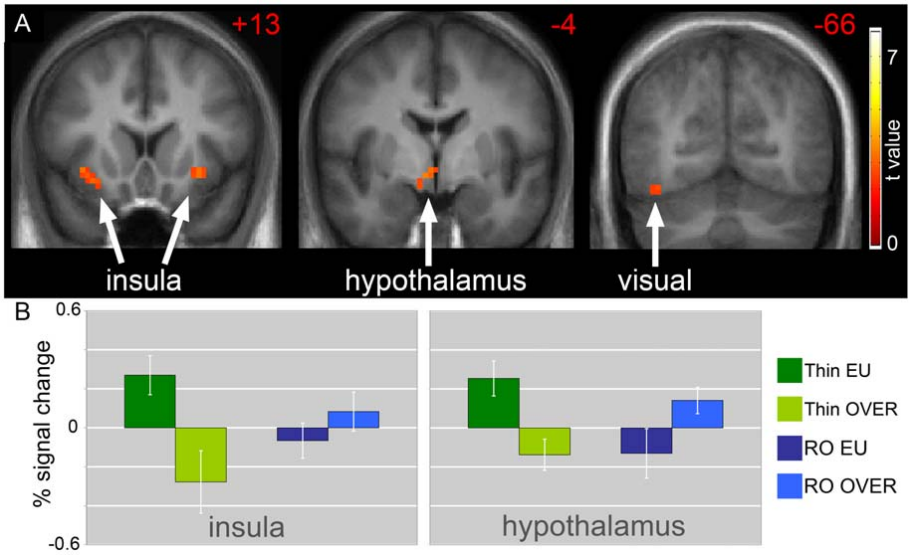
Effects of overfeeding on the neuronal response in thin as compared to reduced-obese individuals. The difference in neuronal response with overfeeding as compared to eucaloric feeding in thin as compared to reduced-obese individuals in response to foods of high hedonic value is shown (EU>OF:Thin>OF:H>O). A. Greater deactivation of the insula, hypothalamus and visual cortex is noted in thin as compared to reduced-obese individuals. Statistical maps thresholded at p<0.01 for visualization and overlaid onto the group average anatomical image. Data are shown in the radiological convention (right hemisphere on the left). B. Mean BOLD responses (± SEM) are shown for the insula and hypothalamus.

Unlike in the thin cohort, overfeeding did not attenuate the neuronal responses seen in the eucaloric state in RO (RO:EU>OF:H>O). As shown in Figure 3A, overfeeding resulted in significantly greater deactivation of the right insula (t(1,75) = 3.13, p = 0.049), right inferior visual cortex (t(1,75) = 3.08, p = 0.025), and hypothalamus (t(1,75) = 3.47, p = 0.017) in thin as compared to RO individuals (EU>OF:Thin>RO:H>O). In other words, overfeeding did not attenuate the neuronal responses to food cues in these brain regions in RO individuals. An additional exploratory whole-brain analysis using a statistical threshold of p<0.005, uncorrected, showed that insular and visual cortex differences to be bilateral and additional differences in the hippocampus and inferior prefrontal cortex. Figure 3B shows neuronal responses for local maxima in the insula and hypothalamus, in terms of BOLD% signal change, relative to the global mean. These data suggest that differences are not solely driven by reduced responses to overfeeding in the RO cohort. Overfeeding in this group was associated with a trend towards increased activation of the hypothalamus (t(1,75) = 2.50, p = 0.1) (OF>EU:H>O).

### Measures of Appetite

Overfeeding resulted in significant reductions in pre-meal hunger (78.8±2.2 to 71.2±2.5 mm, p = 0.009) and prospective food consumption ratings (79.3±2.2 to 71.0±2.4 mm, p = 0.003) and significant increases in post-meal satiety ratings (75.1±2.9 to 82.3±1.8, p = 0.01) as measured by visual analog scales although no group effects were seen. Changes in pre-meal hunger and prospective food consumption with overfeeding correlated with changes in insular activation with overfeeding (t = 1.33, p = 0.09 and t = 2.00, p = 0.03, respectively).

## Discussion

The present study was performed to examine the central response to food-related visual cues during states of energy balance and short-term positive energy balance in thin individuals screened to be resistant to weight gain and obesity as compared to reduced-obese (RO) individuals, who are prone to weight gain/regain. The results of this study demonstrate that during energy balance, food-related food cues result in activation of brain regions known to be important in energy intake regulation in both study groups. Thin individuals, however, have a more robust response to food-related visual cues than RO individuals. In addition, thin individuals appear to be more sensitive to the positive energy balance associated with overfeeding with significant attenuation of the neuronal responses to visual food cues as compared to RO individuals.

In the overnight fasted state, the neuronal response to food-related visual cues as compared to non-food objects is complex, associated with the activation of a network of brain regions, including the insula, inferior temporal visual cortex, posterior parietal cortex, ventral striatum, posterior cingulate, hippocampus, sensory cortex, and lateral prefrontal cortex. The activation of a number of these regions is consistent with increased attention to food cues and enhanced motivation to eat. Interestingly, thin individuals appear to be more sensitive to food cues than RO individuals as demonstrated by increased activation of the insula and visual cortex. While we are not aware of any other published study that has examined the difference in response to visual food cues between normal weight and reduced-obese or obese individuals, studies examining regional cerebral blood flow (rCBF) as measured by positron emission tomography (PET) also have shown that obese and RO individuals appear to have altered brain responses to fasting [26], [27], [28], [29]. While Rosenbaum et al showed that the reduced-obese state was associated with significantly greater activation of the brainstem, parahippocampus, culmen, globus pallidus, middle temporal gyrus, inferior frontal gyrus, middle frontal gyrus and lingual gyrus and significantly reduced activation of the hypothalamus, amygdala, parahippocampus, cingulate, hippocampus, middle frontal gyrus, inferior parietal lobule, fusiform gyrus, supramarginal gyrus and precentral gyrus in response to visual food cues as compared to the obese state they did not compare these states to normal weight individuals [30]. We interpret our findings to mean that in the fasted or “hungry” state, obese resistant individuals are more sensitive to food cues, promoting enhanced attention toward food and motivation to eat perhaps in defense of their lower body weight or energy stores.

Two days of positive energy balance as produced by 30% overfeeding has a much more dramatic impact on the neuronal response to visual food cues in thin as compared to RO individuals. Similar to our previous report [6], overfeeding results in diminished activation in cortical regions associated with visual processing and motivation, suggesting that the salience of the food cues is reduced after overfeeding in thin individuals. In addition, reduced hypothalamic activation in response to overfeeding may reflect interactions between visual cues and the energy status of the individual. In a state of positive energy balance the “gain” on the homeostatic regulation of energy balance may be changed, promoting reduced salience to food related cues and a return to energy balance. In contrast, RO individuals have increased activation of cortical regions associated with visual processing and attention as well as in the hypothalamus, suggesting altered ability to sense positive energy balance. This altered response in RO individuals signifies an impaired interaction between visual food cues and brain regions important in the regulation of food intake and may represent a potential mechanism for explaining the difficulty individuals have with maintaining weight loss.

While we are not aware of any other studies that have examined the effects of positive energy balance on the neuronal response to visual food cues, the neuronal responses to food-related stimuli have been shown to be affected by acute satiation, supporting the concept that the metabolic state effects the response and processing of external food-related stimuli [14], [15], [31], [32], [33]. In addition, acute satiation has been shown to be associated with increased rCBF in the prefrontal cortex and decreased rCBF in the hypothalamus and insula [34], [35]. It may be that the changes in corticolimbic activation with alterations in energy state drive the hypothalamic response, or it may be that the hypothalamus integrates homeostatic and nonhomeostatic signals directly [9], [36]. Again, this signaling appears to be altered in RO individuals who are prone to weight gain.

The findings of the present study and of others suggest that the insula plays a central role in the response to food stimuli. Although usually considered the primary taste cortex, the insula has also been shown to be a brain region important in the regulation of feeding behaviors [31], [37], [38] and may relate to the memory of the rewarding effects of food [39], [40]. The fasted or ‘hungry’ state is consistently associated with not only increased rCBF in the insula [27], [34], [41] but also increased activation of the insula in response to visual food-related stimuli [6], [11], [12], [13], [14], [16], [17], [38], [42], [43], and insular activation in response to food cues has also been found to be correlated with the desire to eat [11] as well as to hunger and prospective food intake as seen in our current findings. Satiation, on the other hand, is associated with reduced insula rCBF, and overfeeding results in the attenuation of the insula in response to visual (current findings) and olfactory stimuli [20]. It, therefore, appears that visual food cues may be associated with activation of the memory of the rewarding effects of food and goal directed behavior potentially preparing the individual for ingestion, and that these signals are ‘normally’ turned off in times of positive energy balance. The insular response to food cues, however, appears to be altered in RO individuals who do not appear to appropriately “turn off” the insula in response to overfeeding.

The insula has also been shown to be important in somatosensory, visceral sensory, and visceral motor functions, such as in response to esophageal stimulation and gastric distension [28], [37], [44], [45]. Furthermore, peripheral signals such as leptin and ghrelin have been found to impact the insular response to food stimuli. In leptin-deficient adults, the leptin deficient state is associated with greater activation of the insula in response to visual stimuli of high-calorie foods than during leptin replacement. These authors concluded that “these findings may reflect the role of the insula in representing information about the internal bodily states as conscious emotional feelings, or interoception. Leptin deficiency may enhance insular interoception of cue induced feelings of hunger” [46]. Leptin replacement in reduced obese individuals is also associated with reduced insular activation in response to visual food stimuli [30]. Malik et al found that ghrelin administration was associated with increased activation of insula in response to visual food stimuli [47]. It is unclear, however, how these peripheral signals mediate activation of the insula. In addition to the hypothalamus, receptors for these hormones have been found in the cerebral cortex, hippocampus, basal ganglia, brainstem, and cerebellum [48], but it is not known whether there are receptors for these hormones in the insula specifically. Although an indirect effect is possible, the hypothalamus is a likely region mediating the response seen in the insula. These findings support that the insula is a brain region that is important in the processing of food-related cues, both internal and external, and appears to be important in processing the motivational value of food and feeding.

In conclusion, the results of this study demonstrate that there are important differences in the responses to visual food-related cues between thin individuals, who have been screened to be resistant to weight gain and obesity, and reduced-obese individuals, individuals who are prone to weight gain/regain. In the baseline fasting state, thin individuals have a much more robust neuronal response to food-related visual cues than reduced-obese individuals. Overfeeding results in significant attenuation of the response to visual foods cues in thin but not reduced-obese individuals. These findings emphasize the important role of external visual cues in the regulation of energy intake and suggest that there are important phenotype differences in the interaction between external visual sensory inputs, energy balance status, and brain regions important in the regulation of energy intake.
